# Consensus Recommendations to Optimize Testing for New Targetable Alterations in Non-Small Cell Lung Cancer

**DOI:** 10.3390/curroncol29070396

**Published:** 2022-07-15

**Authors:** Diana N. Ionescu, Tracy L. Stockley, Shantanu Banerji, Christian Couture, Cheryl A. Mather, Zhaolin Xu, Normand Blais, Parneet K. Cheema, Quincy S.-C. Chu, Barbara Melosky, Natasha B. Leighl

**Affiliations:** 1 BC Cancer, Department of Pathology, Vancouver, BC V5Z 4E6, Canada; dionescu@bccancer.bc.ca; 2 Division of Clinical Laboratory Genetics, Laboratory Medicine Program, University Health Network, Toronto, ON M5G 2C4, Canada; tracy.stockley@uhn.ca; 3 Department of Laboratory Medicine and Pathobiology, University of Toronto, Toronto, ON M5S 1A8, Canada; 4 CancerCare Manitoba Research Institute, Winnipeg, MB R3E 0V9, Canada; sbanerji@cancercare.mb.ca; 5 Department of Internal Medicine, Rady Faculty of Health Sciences, University of Manitoba, Winnipeg, MB R3A 1R9, Canada; 6 Service of Anatomic Pathology and Cytology, Institut Universitaire de Cardiologie et de Pneumologie de Quebec-Université Laval, Québec City, QC G1V 4V5, Canada; christian.couture.med@ssss.gouv.qc.ca; 7 Department of Laboratory Medicine and Pathology, Faculty of Medicine & Dentistry, Edmonton, AB T6G 2B7, Canada; cheryl.mather@albertaprecisionlabs.ca; 8 Department of Pathology, QE II Health Sciences Centre, Halifax, NS B3H 1V8, Canada; zxu3@dal.ca; 9 Department of Pathology, Dalhousie University, Halifax, NS B3H 4R2, Canada; 10 Hematology-Oncology Service, Department of Medicine, Centre Hospitalier de l′Université de Montréal, 1051, Rue Sanguinet, Montreal, QC H2X 3E4, Canada; normand_blais@hotmail.com; 11 William Osler Health System, University of Toronto, Brampton, ON L6R 3J7, Canada; parneet.cheema@williamoslerhs.ca; 12 Division of Medical Oncology, Department of Oncology, Cross Cancer Institute, University of Alberta, Edmonton, AB T6G 1Z2, Canada; quincy.chu@albertahealthservices.ca; 13 BC Cancer–Vancouver Centre, Vancouver, BC V5Z 4E6, Canada; bmelosky@bccancer.bc.ca; 14 Princess Margaret Cancer Centre, University of Toronto, Toronto, ON M5G 2C1, Canada

**Keywords:** targeted therapy, NSCLC, biomarker, genomic profiling, next-generation sequencing

## Abstract

Non-small cell lung cancer (NSCLC) has historically been associated with a poor prognosis and low 5-year survival, but the use of targeted therapies in NSCLC has improved patient outcomes over the past 10 years. The pace of development of new targeted therapies is accelerating, with the associated need for molecular testing of new targetable alterations. As the complexity of biomarker testing in NSCLC increases, there is a need for guidance on how to manage the fluid standard-of-care in NSCLC, identify pragmatic molecular testing requirements, and optimize result reporting. An expert multidisciplinary working group with representation from medical oncology, pathology, and clinical genetics convened via virtual meetings to create consensus recommendations for testing of new targetable alterations in NSCLC. The importance of accurate and timely testing of all targetable alterations to optimize disease management using targeted therapies was emphasized by the working group. Therefore, the panel of experts recommends that all targetable alterations be tested reflexively at NSCLC diagnosis as part of a comprehensive panel, using methods that can detect all relevant targetable alterations. In addition, comprehensive biomarker testing should be performed at the request of the treating clinician upon development of resistance to targeted therapy. The expert multidisciplinary working group also made recommendations for reporting to improve clarity and ease of interpretation of results by treating clinicians and to accommodate the rapid evolution in clinical actionability of these alterations. Molecular testing of all targetable alterations in NSCLC is the key for treatment decision-making and access to new therapies. These consensus recommendations are intended as a guide to further optimize molecular testing of new targetable alterations.

## 1. Introduction

Lung cancer is the most commonly diagnosed cancer globally and in Canada, excluding non-melanoma skin cancer, and is the leading cause of cancer deaths. Approximately 29,600 Canadians will be diagnosed with lung cancer in 2021, estimated to account for 25% of all cancer deaths [1]. Although advances in research, screening protocols, and targeted therapies have improved mortality from lung cancer in recent years, the 5-year survival rate is still poor at 22% in Canada and lower than 20% in many countries [1].

In the past decade, evolving knowledge of predictive biomarkers has created new therapeutic opportunities for NSCLC [2,3]. It is estimated that 35–50% of patients with advanced non-squamous NSCLC harbor a targetable alteration [3,4,5], and selection of patients based on predictive biomarkers is associated with improved survival and better quality of life [5,6,7,8,9]. Molecular testing of sensitizing *EGFR* mutations, *BRAF* V600E, as well as *ALK*, *ROS1*, and *NTRK* fusions, is now standard-of-care for patients with advanced NSCLC [10], as is testing for the *EGFR* T790M mutation upon resistance to first- or second-generation EGFR tyrosine kinase inhibitor therapy [11,12,13]. Routine testing of *RET* fusions and *MET* exon 14 skipping mutations should also be considered standard-of-care based on the recent Health Canada approvals of targeted therapies for these alterations, supported by current treatment guidelines for advanced lung cancer from the American Society of Clinical Oncology (ASCO) and Cancer Care Ontario (CCO) [13]. New targetable alterations are continuing to emerge, and the pace of lung cancer drug development and new drug approvals is accelerating, with 23 new approvals in the past 5 years compared to 8 drug approvals in the previous 10 years [14]. New targeted therapies for NSCLC that have been recently approved or are under review include amivantimab for *EGFR* exon 20 insertion mutations, sotorasib for *KRAS* G12C, selpercatinib and praseltinib for *RET* fusions, capmatinib and tepotinib for *MET* alterations, and trastuzumab deruxtecan for *HER2* insertion mutations [15,16,17,18,19,20,21]. In addition, new resistance biomarkers are emerging that are relevant for molecular testing upon progression of targeted therapy, such as *ALK* G1202R, *EGFR* C797S, and other resistance alterations in *EGFR*, *ALK*, and *ROS1*, as well as *BRAF*, *MET*, and *NRG1* gene rearrangements [22,23]. Although the incidence of *EGFR, BRAF*, and *MET* exon 14 skipping mutations is significantly lower (approximately 5%, <1%, and 1%, respectively) in squamous cell carcinoma (SCC) [3], these alterations are still relevant to patient treatment if detected. Certain clinical characteristics such as young age or lack of smoking history can enrich a subset of patients with SCC with a higher likelihood of an oncogenic driver mutation, but will not identify all cases. In addition, alterations in *FGFR1*, *FGFR2*, and *FGFR3* occur in SCC and may soon be targetable with FGFR inhibitors [24,25,26,27]. Other predictive biomarkers used in NSCLC management include PD-L1 expression for pembrolizumab therapy [28].

To ensure that patients receive the most appropriate treatment, broad upfront molecular testing needs to include both established and new targetable alterations. Here, we use a comprehensive panel that includes new targetable alterations for molecular testing that benefits patients by facilitating access to novel treatments as part of standard-of-care, or through clinical trials and compassionate programs, as well as supporting adoption of new targeted therapies into routine care. Treatment algorithms and guidelines including novel targeted therapies at diagnosis and progression are rapidly evolving, creating a need for guidance on how to address the fluid standard-of-care in NSCLC, pragmatic molecular testing requirements, and steps to optimize result reporting. To address this need, a national expert multidisciplinary working group was convened to discuss recommendations to help oncologists and patients with decision-making when considering molecular testing in NSCLC, and to help clinical laboratories optimize delivery of such testing.

## 2. Methods

An expert multidisciplinary working group was formed to develop recommendations for testing of new targetable alterations in NSCLC. The group had pan-Canadian representation and included medical oncologists, pathologists, and a clinical geneticist. A targeted literature review was performed to identify relevant literature and inform the recommendations. The expert multidisciplinary working group convened in two virtual meetings to discuss draft recommendations. Following the virtual meetings, the recommendations were revised and reviewed again by all working group members to reach a final set of consensus recommendations. Patient advocates were also invited to review the recommendations and manuscript and participate in the development of a plain language infographic.

## 3. Results and Discussion

### 3.1. Biomarker Testing for New Targetable Alterations

To support accurate and timely testing for new targetable alterations, the expert multidisciplinary working group developed consensus recommendations that address foundational issues in testing (Table 1). For the purposes of these recommendations, the expert multidisciplinary working group defined new targetable alterations as those associated with clinical benefit (or lack thereof) with a targeted therapy alone or in combination with other therapies (strength of support: Phase III, II, I studies and case reports) or being studied in advanced stages of clinical development. Other biomarkers are emerging that may be relevant for selection of immunotherapies such as co-alterations in *TP53*, *STK11*, and *KEAP1* genes; however, the working group considered those to be outside of the scope of these recommendations.

The patient advocates who reviewed the consensus recommendations felt that the recommendations were clear and if followed, would help optimize patient care. They identified key elements of the work to highlight for patients, which were then incorporated into a plain language infographic (Recommendations for Comprehensive Biomarker Testing in Advanced Non-Small Cell Lung Cancer, Supplementary Figure S1).

### 3.2. Foundational Recommendations

#### 3.2.1. New Targetable Alterations as Part of a Comprehensive Biomarker Panel

The detection of all potential targetable alterations as part of upfront molecular testing is critical for optimal treatment decision-making; therefore, the expert multidisciplinary working group recommends that all targetable alterations, including new targetable alterations as well as historical standard-of-care targetable alterations, be tested using a comprehensive next-generation sequencing panel. The use of comprehensive gene panels for biomarker testing in advanced lung cancer is supported by international guidelines, including National Comprehensive Cancer Network (NCCN), European Society for Molecular Oncology (ESMO), and College of American Pathologists-International Association for the Study of Lung Cancer-Association for Moecular Pathology (CAP-IASLC-AMP) [9,29,30,31]. The inclusion of new targetable alterations as part of a comprehensive gene panel also aligns with current Canadian consensus recommendations to include new and emerging actionable targets on comprehensive gene panels to identify patients for standard therapies, clinical trials, or early drug access programs [12]. Due to advancements in technology, the increased cost of a comprehensive panel that includes new targetable alterations compared to historical biomarker panels is modest, but comprehensive panels provide additional clinically relevant information that is essential to optimal treatment decisions. For example, in one study, a comprehensive panel identified additional actionable alterations in 31% of patients with stage IV NSCLC, and additional clinical trial options in 75% of patients [32]. The adoption of an even broader panel of genes beyond what is outlined in this publication is also possible from a financial and technical perspective. Comprehensive panels are preferred over single-gene testing because they allow concurrent testing of all relevant biomarkers, which saves time and make efficient use of the tissue specimen, as very few patients have enough tissue for serial testing of all relevant biomarkers [33,34,35].

#### 3.2.2. Selection of Molecular Tests to Detect Clinically Relevant Alterations

Although a detailed review of testing methods is beyond the scope of this initiative, a summary of current testing methodologies and their applicability to targetable alterations is provided in Table 2 to assist clinicians and patients, and includes low-throughput (e.g., fluorescence in situ hybridization (FISH), immunohistochemistry (IHC), polymerase chain reaction (PCR)) and high-throughput methods (e.g., comprehensive NGS). The selection of appropriate molecular tests is critical for optimal detection of emerging and established biomarkers and associated treatment decisions. It is critical that the molecular tests used can detect all alterations that are clinically relevant in a specific gene, including gene fusions, copy number variants, single nucleotide variants, and small insertion/deletions. In addition, it is important to understand and consider the limitations within a given assay. For example, EGFR exon 20 insertions (Exon20ins) are molecularly heterogeneous and account for approximately 5% of EGFR mutations in NSCLC in Canada [36], and up to 10% of EGFR mutations in NSCLC internationally [37]. The most commonly identified Exon20ins variant is V769_D770insASV, but it accounts for only about 20% of all *EGFR* Exon20ins mutations [37]. In a recent study, 102 unique Exon20ins mutations were identified in the FoundationInsights^TM^ database [38]. As PCR methods are designed to identify only targeted regions of interest, it is estimated that approximately 50% of Exon20ins variants would be undetected by the RT-PCR approach in comparison to a more comprehensive sequencing approach such as NGS [38,39]. Therefore, the clinical utility of molecular tests that laboratories may provide has different applicability and limitations depending on the biomarker: Table 3 provides an overview of the applicability of various molecular tests to new targetable alterations for both tissue and liquid biopsy specimens. 

As comprehensive NGS panels can have the capability to detect fusions, copy number variants, single nucleotide variants, and small insertion/deletions, many laboratories may choose to utilize NGS panel assays. Comprehensive NGS panels provide high sensitivity and the ability to assess multiple types of variants simultaneously, and some integrate both DNA and RNA analysis into a combined workflow. The limitations of individual NGS assays must be considered, in particular, their ability or inability to detect certain alterations. Key points to consider include the targets included in the panel, target enrichment approaches (amplicon versus hybrid-capture library preparations), and whether the sample input is DNA, RNA, or both. Certain NGS panels are limited to the detection of specific targeted regions in a gene (“hotspots”) and thus alterations occurring beyond these regions will not be detected. Two main types of library preparation used for target enrichment in NGS include amplicon and hybrid-capture. The amplicon-based approach relies on primers that flank the regions of interest for sequencing which may lead to false negatives due to allele-dropout or genomic deletions. The hybrid-capture-based method utilizes probes to capture longer segments of the target genome for sequencing, enabling sequencing of regions surrounding the area of interest, and is less prone to allele dropout. However, limitations of the hybrid-capture-based method include potential off-target sequencing, longer workflow, and need for larger input DNA quantities [49]. Panel selection based on target enrichment approaches has been demonstrated to be critical when detecting alterations such as *MET* exon 14 skipping (*MET*ex14) events, as an example. Due to the diversity of mechanisms that can lead to *MET*ex14 events, utilizing an amplicon-based NGS panel results in reduced detection rates due to the diversity of alterations, and thus hybrid-capture has been the preferred approach when performing DNA-only-based testing [50,51]. Parallel or sequential RNA-based testing can aid in identifying *MET*ex14 events by detecting fusions of exons 13 and 15. Clinical laboratories using NGS assays, clinicians, and patients, where possible, should be aware of the limitations of the assays in the detection of new targetable alterations (Table 4).

#### 3.2.3. Timing of Comprehensive Biomarker Testing

The expert multidisciplinary working group recommends that comprehensive biomarker testing including new targetable alterations should be initiated by the pathologist at the time of diagnosis of non-squamous NSCLC as a reflex test. Comprehensive biomarker testing should also be considered beyond adenocarcinoma for patients that may have an enhanced incidence of driver mutations, e.g., testing for MET alterations in pulmonary sarcomatoid carcinomas. For patients with non-squamous NSCLC, reflex testing should be initiated regardless of disease stage, as the use of targeted therapies is no longer limited to patients with advanced disease [69]. With many trials currently underway evaluating the use of targeted therapy in the adjuvant setting, there will be increasing use of targeted therapies in earlier stages of disease. Reflex testing at the time of diagnosis helps optimize time to treatment, allowing for molecular testing to be initiated before the first oncology consultation and increasing the chance of having molecular results available to the oncologist at the time of the initial consultation. Reflex testing has been shown to significantly improve time to treatment and has also been associated with improved progression-free survival rates in patients with *EGFR*-mutated NSCLC treated with EGFR TKIs [70,71,72,73].

#### 3.2.4. Liquid Biopsy as a Complementary or Alternative Approach for Molecular Profiling

Liquid biopsy may be used as a complementary or alternative approach to tissue-based genomic profiling in patients with advanced NSCLC [74]. In a liquid biopsy, cell-free DNA (cfDNA), which includes circulating tumor DNA (ctDNA) shed by the tumor, is isolated from a peripheral blood sample and used for biomarker testing. In addition to detection of small mutations and insertions/deletions, selected liquid biopsy assays have been demonstrated to detect fusions such as *RET*, as well as *MET* fusions resulting from exon 14 skipping mutations and *MET* amplification [75,76,77,78,79]. In addition, liquid biopsies have been used as an alternative for patient selection in registration trials of new targeted therapies, including the detection of *RET* fusions for pralsetinib therapy and the detection of *MET* exon 14 skipping mutations for tepotinib therapy [19,20]. Liquid biopsy offers potential advantages over tissue biopsy for molecular profiling, including faster turnaround time, a less invasive procedure for the patient, and overcoming issues with tumor heterogeneity [74]. However, liquid biopsy has limitations, in particular, that low levels of circulating tumor DNA (ctDNA) shedding from some tumors can result in low clinical sensitivity [74]. In addition, optimization of preanalytical variables (i.e., sample collection and processing) is critical for successful ctDNA analysis [80,81]. Overall, for NSCLC patients who do not receive reflex tissue genomic profiling, and who have sufficient burden of disease to detect plasma ctDNA, using liquid biopsy to rule in actionable alterations can result in faster turnaround time, reduced costs, and shorter time to targeted treatment [82]. A targeted alteration detected at liquid biopsy can be considered actionable, but a negative result should be confirmed with a tumor tissue biopsy. It is important for clinicians and patients to realize that the same caveats with tissue genomic testing also apply to cell-free DNA panels, i.e., targets identified, sensitivity and specificity parameters, DNA/RNA input, as well as the importance of correction for germline or non-tumoral variants (clonal hematopoiesis) [74].

Upon development of resistance to targeted therapy in patients with advanced lung cancer, comprehensive genomic profiling for all targetable alterations should be performed. Relevant biomarkers for resistance are continuing to emerge: at progression, a significant number of genetic alterations detected are actionable, and approximately another 15% allow for patient enrolment in clinical trials [83,84]. Liquid biopsy is preferred over tissue biopsy at progression as a first step because of its quick turnaround time and ability to provide information about the complete mutational landscape of the cancer [74]. Comprehensive biomarker testing upon development of resistance to targeted therapy is a necessary part of the care pathway for some patients, and therefore those patients may require genomic profiling using an NGS assay both at diagnosis and at progression.

#### 3.2.5. Implementation of Molecular Tumor Boards

With the complexity of the biomarker landscape and access to new targeted therapies in NSCLC, molecular tumor boards can be a helpful resource in clinician education about genomic medicine. Molecular tumor boards provide support to clinicians and their patients to gain a greater understanding of the functional impact of genomic alterations identified, relevant therapies, and even clinical trials that may be of value for patients [85,86]. The expert multidisciplinary working group therefore recommends that molecular tumor boards should be established provincially and within institutions to aid in interpretation of complex genomic results and selection of appropriate therapy, preferably integrated into existing tumor-specific multidisciplinary case conferences or tumor boards.

### 3.3. Interpretation and Reporting of New Targetable Alterations

The expert multidisciplinary working group also developed consensus recommendations for the interpretation and reporting of new targetable alterations (Table 5).

#### 3.3.1. Laboratory Accreditation

With the rapid evolution and testing capabilities of targetable alterations, participation in external quality assessment (EQA) programs is critical and ensures that quality of biomarker testing is maintained [87,88]. Laboratories must be accredited to perform clinical testing as required by their jurisdiction in Canada and must participate in external quality control programs. The expert multidisciplinary working group recommends that laboratories should monitor the rates of positivity and assay failure for each biomarker in the comprehensive panel at the frequency required by their provincial or regional laboratory accreditation programs.

#### 3.3.2. Key Elements on a Clinical Report

With the ongoing addition of new clinically relevant biomarkers, the complexity of molecular reports in NSCLC is increasing. Clinicians and patients need to be able to discern the key actionable results from the large amounts of data presented in the molecular report. The expert multidisciplinary working group recommends that molecular reports should have a clear, top line summary of the key, clinically relevant findings at the beginning of the report. A summary of suggested report content can be found in Supplemental Data (Summary of Report Content, Supplemental Data S1).

Existing guidelines should be followed for the reporting of new targetable alterations [89,90,91]. The expert multidisciplinary working group highlighted elements of reporting that are of particular importance in the context of new targetable alterations. The working group recommends that laboratories should assess tumor cellularity, and that the quality and quantity of DNA and/or RNA be assessed and documented prior to biomarker testing. As specimens may sometimes contain multiple blocks or sections, or the same patient may have multiple biopsy specimens from different institutions, the molecular report should state which specimen and block were tested. In the case of test failure, an attempt should be made first by the pathologist and then by the clinical team to identify another specimen for testing. Describing the molecular testing method in the report is critical for accurate interpretation of test results, and can also enable identification of samples that could benefit from reanalysis when updates to methodology allow for detection of additional alterations [92]. In addition, molecular reports should contain a description of the methodology used, and limitations of assays used to detect targetable alterations should be clearly communicated, as required by laboratory accreditation programs and recommended in international guidelines [91]. This is particularly important in the context of new targetable alterations, where oncologists may not be aware of the limitations of the assays that laboratories are using to detect these alterations.

#### 3.3.3. Variant Classification System

The report should classify alterations detected using a standardized tier system, such as that described by AMP/ASCO/CAP for the interpretation and reporting of sequence variants in cancer [90], and should state the tier of each reported variant. Tier I and II variants, representing variants of strong clinical significance and potential clinical significance, respectively, should be included in the top line summary of clinically relevant findings. Laboratories may choose to pool tier I and II variants in their reports as tier I/II. Tier III variants of unknown significance should also be included in the report, since emerging evidence may change the actionability of variants over time. Tier IV alterations (variants deemed benign or likely benign) should not be listed in the report. At the request of the treating oncologist, laboratories should reevaluate previous reports for changes in actionability of reported alterations. When an alteration changes in clinical significance and actionability, in discussion with the clinician, the laboratory should consider issuing a revised report. The expert multidisciplinary working group suggests that laboratories should participate in external training programs for interpretations of new targetable alterations in NSCLC.

#### 3.3.4. Variant Interpretation in the Context of Clinical Significance

With the increasing number of biomarkers that are clinically relevant for treatment decisions in NSCLC, the report is an important information resource regarding the variants detected by the comprehensive molecular profiling. The laboratory team should ensure that the information provided in the report is as up-to-date as possible. Variants in the report should be described at the cDNA and protein level, using Human Genome Variation Society (HGVS) nomenclature as recommended by international guidelines [90]. In addition, if needed for ease of interpretation by treating clinicians, nomenclature associated with treatment indications should be included, as agreed upon by the multidisciplinary clinical team. For example, the commonly recognized nomenclature *EGFR* T790M should be used, as well as the HGVS nomenclature NM_005228.4(EGFR):c.2369C > T(p.Thr790Met). Variant annotation should include a description of the protein, the variant type, exon location if clinically relevant, and a brief summary of the clinical importance of the variant including expected responsiveness/resistance to therapies from reputable sources. The inclusion of new targetable alterations as part of comprehensive biomarker panel testing is helpful for determining clinical trial eligibility and early access programs, and thus all Tier I to III variants should be reported. As it is very challenging for reports to contain relevant and up-to-date current clinical trials for which a patient may be eligible, specific clinical trial recommendations are not recommended for inclusion. However, the expert panel recommends that reports refer to resources for finding such information, to aid the oncologist in making treatment decisions. Examples include www.clinicaltrials.gov, www.canadiancancertrials.ca in Canada, and other local resources relevant to each region.

## 4. Conclusions

With the increasing pace of development of new targeted therapies for NSCLC, comprehensive molecular testing of all targetable alterations is critical to ensure that patients receive the most appropriate care. The consensus recommendations herein aim to provide guidance to laboratories, pathologists, and oncologists on how to optimize molecular testing for new targetable alterations and manage the evolving standard-of-care in NSCLC.

## Figures and Tables

**Table 1:** Summary of foundational recommendations.

**I**	**New Targetable Alterations as Part of a Comprehensive Biomarker Panel** - All targetable alterations should be tested as part of a comprehensive panel that includes the standard-of-care biomarkers as summarized by current Canadian consensus recommendations as well as international guidelines, including the National Comprehensive Cancer Network, College of American Pathologists, the International Association for the Study of Lung Cancer, and the Association for Molecular Pathology.
**II**	**Selection of Molecular Tests to Detect Clinically Relevant Alterations** - Molecular tests used should be able to detect all mutation types relevant for targetable alterations, including gene fusions, copy number variants, single nucleotide variants, and small insertion/deletions.
**III**	**Timing of Comprehensive Biomarker Testing** - Comprehensive biomarker testing, including new targetable alterations, is recommended for all patients diagnosed with non-squamous NSCLC and should be initiated by the pathologist at the time of initial diagnosis as a reflex test. Upon analytic failure due to insufficient nucleic acid content, other techniques such as single gene assays should be attempted if no other sample is available. - Comprehensive biomarker testing, including new targetable alterations, should also be considered beyond adenocarcinoma for patients that may have an enhanced incidence of driver mutations, and when treatment can be impacted by the results of testing. - Comprehensive biomarker testing for all targetable alterations should be performed at the development of resistance to targeted therapy in patients with advanced NSCLC.
**IV**	**Liquid Biopsy as a Complementary or Alternative Approach for Molecular Profiling** - Liquid biopsy * can be considered as an alternative or complementary approach to tissue genomic testing in patients with advanced NSCLC. - Liquid biopsy is preferred over tissue biopsy as a first step at progression after a targeted therapy for identification of mechanisms of acquired resistance to targeted therapies. - A targeted alteration detected at liquid biopsy can be considered actionable, but a negative result should be confirmed with a tumor tissue biopsy.
**V**	**Implementation of Molecular Tumor Boards** - Molecular tumor boards ** should be established provincially and within institutions to aid in interpretation of results and selection of appropriate therapy.

* Testing of biomarkers from a peripheral blood sample. ** Molecular tumor boards are multidisciplinary groups of experts that can provide support to clinicians and their patients to gain a greater understanding of the functional impact of targetable alterations identified, as well as possible therapies and clinical trials.

**Table 2:** Methodologies for testing of targetable alterations in NSCLC.

Test	Variant Detection	Limitations
**Fluorescence in Situ Hybridization (FISH)** [40]	Translocations, large deletions, and duplications/amplifications	-May not be informative regarding specific fusion partners
**Immunohistochemistry (IHC)** [41]	Protein expression	-May require additional confirmatory molecular/cytogenetic testing (e.g., *ROS1*)
**Polymerase Chain Reaction (PCR) Methodologies**		
**Endpoint PCR** [42]	SNVs, small insertions/deletions, splice variants (at exon boundaries)	-Limited ability to detect translocations and large deletions
**Reverse Transcription PCR (RT-PCR)** [43]	SNVs, small insertions/deletions, splice variants, and fusions	-Inability to detect fusions with novel partners -Usually limited to known variants and established break points
**Droplet Digital PCR** [44]	SNVs, small insertions/deletions, splice variants (at exon boundaries)	-Limited ability to detect translocations and large deletions -Potential for multiplex detection of several biomarkers is limited
**Next-Generation Sequencing (NGS) Methodologies** [45]		- Requires NGS platform equipment and skill with bioinformatics tools -Variants observed may be of uncertain clinical significance
**DNA-Based** [46]	Typically used to detect SNVs, CNVs, small insertions/deletions; can be customized to detect gene fusions	-Addition of gene fusion coverage may impair overall assay sensitivity and increase cost
**RNA-based** [47]	Typically used to detect fusions but can also be used to detect SNVs, CNVs, small insertions/deletions	-Theoretically may be impacted by quality of RNA especially from older FFPE material
**Amplicon-Based Library** [48]	SNVs, CNVs, small insertions/deletions, fusions, and splice variants	-Panel will only detect targets included in the amplified regions, so sensitivity may be reduced for some variants
**Hybrid Capture-Based Library** [48]	SNVs, CNVs, small insertions/deletions, fusions, and splice variants	-More time-intensive than amplicon-based NGS -Requires larger amounts of input DNA compared to amplicon-based NGS

**Table 3:** Molecular testing techniques for targetable alterations in NSCLC.

	Variant Type	Tissue Biopsy Specimens				Liquid Biopsy Specimens	
		IHC	FISH ^1^	PCR ^2^	NGS ^3^	PCR ^2^	NGS ^3^
**Established Targets**	*EGFR* mutations (sensitizing and T790M)	—	—	••	••	•• ^4^	••
	*ALK* fusions ^5^	••	••	•	••	•	••
	*ROS1* fusions	S	••	•	••	•	••
	*NTRK* fusions	S	••	•	••	—	•
	*BRAF* mutations	• ^6^	—	••	••	••	••
**Updated Target Inclusion**	*EGFR* exon 20 insertions	—	—	•	••	•	••
	*EGFR* resistance mutations (excluding T790M)	—	—	••	••	••	••
	*MET* exon 14 skipping mutations	—	—	••	••	•	•
	*KRAS* G12C mutation	—	—	••	••	••	••
	*HER2* mutations	—	—	••	••	•	••
	*RET* fusions	—	••	•	••	•	•
	*MET* amplification	— ^7^	••	—	••	—	•
	*ALK* mutations	—	—	••	••	•	••
	*ROS1* mutations	—	—	••	••	•	••
	*BRAF* fusions	—	••	—	••	—	•
	*MET* fusions	—	••	—	••	—	•
	*NRG1* fusions	— ^7^	••	—	••	—	•

^1^ Limitations of FISH for detection of targetable alterations in NSCLC: FISH using break-apart probes is not informative regarding the specific fusion partner. ^2^ Limitations of PCR for detection of targetable alterations in NSCLC: for fusions, PCR will not detect unknown or novel fusion partners. For *EGFR* exon 20 insertion mutations, PCR only detects a small number of the known insertion mutations. ^3^ Limitations of NGS for detection of targetable alterations in NSCLC: the capability and sensitivity of NGS assays to detect specific variants depends on the details of the NGS assay used and the input nucleic acids, but high sensitivity detection of all targetable alterations is possible to achieve. ^4^ ddPCR is a suitable method for detecting *EGFR*-sensitizing mutations, *EGFR*-T790M, and *KRAS* G12C. ^5^ Oncogenic fusions resulting from gene rearrangements. ^6^ For V600E variant.^7^ IHC assays are in development. —: not useful. S: screening test only: IHC assays can be used as a screening tool, but a positive result is not definitive and needs to be confirmed with another method. •: lower clinical utility. ••: higher clinical utility.

**Table 4:** Capabilities of selected NGS assays for detection of targetable alterations in NSCLC.

Assay Name	Nucleic Acid Input	Target Enrichment Method	Platform	Ability to Detect New Targetable Alterations	Number of Genes/Targets	Variant Type Detection
**Oncomine Precision Assay** [52]	DNA, RNA, or cfDNA	amplicon-based	Ion Torrent Genexus Integrated Sequencer	yes	50 genes; 45 hotspot, 14 CNV genes, 16 intergenetic fusions, 3 intragenetic fusions	SNVs, indels, CNVs, fusions
**Oncomine Focus Assay** [53]	DNA, RNA	amplicon-based	Ion GeneStudio S5, S5 Plus, or S5 Prime System	yes, except *NRG1* fusion	52 genes; 35 hotspot regions, 19 copy number genes, 23 fusions	SNVs, indels, CNVs, fusions
**Oncomine Lung cfDNA Assay** [54]	cfDNA	amplicon-based	Ion GeneStudio S5 System, Ion PGM System, Ion S5 XL System	yes, except fusions and amplifications	11 genes; >150 hotspot regions	SNVs, indels
**Oncomine Comprehensive Assay v3** [55]	DNA, RNA	amplicon-based	Ion Torrent Genexus System, Ion GeneStudio S5 System	yes	161 genes; 87 hotspot regions, 43 focal CNV gains, 48 full CDS for del mutations, 51 fusion drivers	SNVs, indels, CNVs, fusions
**Oncomine Comprehensive Assay Plus** [56]	DNA, RNA	amplicon-based	Ion GeneStudio S5 Prime System, Ion GeneStudio S5 Plus System	yes	>500 genes; 165 hotspot regions, 333 genes with focal CNV gains/loss, 227 full CDS, 49 fusion driver genes	SNVs, indels, CNVs, fusions, TMB, MSI
**QIAseq Pan-Cancer Multimodal Panel** [57]	DNA, RNA	amplicon-based (simultaneous DNA, RNA enrichment)	MiniSeq, MiSeq, NextSeq 500/550, HiSeq 2500, HiSeq 3000/4000, and NovaSeq 6000	yes	523 genes; panel size of 1.44 Mb, 56 fusions, 26 MSI loci	SNVs, indels, CNVs, fusions, gene expression, TMB, MSI
**AmpliSeq for Illumina Cancer Hotspot Panel v2** [58]	DNA	amplicon-based	iSeq 100, MiniSeq, MiSeq	can only detect hotspot SNVs and indels in *EGFR, KRAS, ERBB2, ALK* genes	Hotspot regions across 50 genes	SNVs, indels
**AmpliSeq for Illumina Focus Panel** [59]	DNA, RNA	amplicon-based	iSeq 100, MiniSeq, MiSeq	yes, except *NRG1* fusion	Biomarkers across 52 genes	SNVs, indels, CNVs, fusions
**AmpliSeq for Illumina Comprehensive Panel v3** [60]	DNA, RNA	amplicon-based	NextSeq 1000, NextSeq 2000, NextSeq 550	yes	161 genes; 86 hotspot regions, 48 full-length genes, copy number genes, and inter- and intragenic gene fusions	SNVs, indels, CNVs, fusions
**AmpliSeq for Illumina Comprehensive Cancer Panel** [61]	DNA	amplicon-based	NextSeq 1000, NextSeq 2000, NextSeq 550	yes, except fusions (i.e., *RET, BRAF, MET, NRG1*)	Full exon coverage of 409 genes	SNVs, indels, CNVs
**TruSight Tumor 15** [62]	DNA	amplicon-based	MiniSeq, MiSeq	can only detect hotspot SNVs and indels in *EGFR, ERBB2, KRAS, MET* genes	Hotspot regions/biomarkers across 15 genes	SNVs, indels
**TruSight Tumor 170** [63]	DNA, RNA	hybrid-based	HiSeq 2500, NextSeq 2000, NextSeq 500, NextSeq 550	yes	Full coding sequences of 170 genes; SNVs and InDels in 151 genes, amplifications in 59 genes, and fusions plus splice variants in 55 genes	SNVs, indels, CNVs, fusions
**TruSight Oncology 500** [64]	DNA, RNA	hybrid-based	NextSeq 500, NextSeq 550, NovaSeq 6000	yes	523 targeted biomarkers aligned with key guidelines and clinical trials; 523 SNVs and indels, 60 focal amp, 55 fusions	SNVs, indels, CNVs, fusions, TMB, MSI
FusionPlex Lung v2 Panel * [65]	RNA	Anchored Multiplex PCR	Illumina^®^ and Ion Torrent™	yes, except *MET* amplification	17 gene targets; 11 SNV/indels, 16 fusions/splicing/exon-skipping	SNVs, indels, fusions
**FusionPlex Pan Solid Tumor v2 Panel** [66]	RNA	Anchored Multiplex PCR	Illumina^®^ and Ion Torrent™	can only detect *MET*ex14 skipping, SNVs in *KRAS, ERBB2*, and *BRAF, MET* and *NRG1* fusions	137 gene targets; 17 SNV/indels, 137 fusions/splicing/exon skipping	SNVs, indels, fusions
**VariantPlex Comprehensive Thyroid and Lung (CTL) Panel** [67]	DNA	Anchored Multiplex PCR	Illumina^®^	yes, except fusions (i.e., *RET, BRAF, MET, NRG1*)	31 gene targets; 29 SNV/indels, 20 CNVs	SNVs, indels, CNVs
**VariantPlex Solid Tumor Panel**	DNA	Anchored Multiplex PCR	Illumina^®^	yes, except fusions (i.e., *RET, BRAF, MET, NRG1*)	67 gene targets; 62 SNVs/indels, 44 CNVs	SNVs, indels, CNVs

* One Canadian laboratory has customized this assay to create a 15-gene RNA panel [68].

**Table 5:** Summary of recommendations for interpretation and reporting of new targetable alterations.

**VI**	**Laboratory Accreditation** - Laboratories should participate in external quality control programs, as well as monitoring the rates of positivity and failure for each biomarker in the comprehensive panel at the frequency required by their laboratory accreditation programs.
**VII**	**Key Elements on a Clinical Report** - Molecular reports should have a clear, top line summary of the key, clinically relevant findings at the beginning of the report. - Tumor cellularity and assessment of the quality and quantity of DNA and/or RNA should be performed and documented prior to biomarker testing. - The molecular report should state which specimen and block were tested. In the case of test failure, an attempt should be made first by the pathologist and then by the clinical team to identify another specimen for testing. - Molecular reports should contain a description of the methodology used, and limitations of assays used to detect targetable alterations should be clearly communicated.
**VIII**	**Variant Classification System** - All detected genetic alterations should be classified using a standardized tier system such as the system recommended by AMP/ASCO/CAP for the interpretation and reporting of sequence variants in cancer: tier I, variants of strong clinical significance; tier II, variants of potential clinical significance; tier III, variants of unknown significance; and tier IV, variants deemed benign or likely benign. Tiers I to III should be included in the report, with the tier of each alteration noted. Tier I and II alterations should be included in the top line summary of clinically relevant findings. Tier IV alterations should not be listed in the report. It is up to the discretion of the reporting facility whether to pool tier I and II variants in their reports as tier I/II. - It is recommended that laboratories reevaluate previous reports for changes in actionability of reported alterations at the request of the treating oncologist. - When an alteration changes in clinical significance and actionability, on request of the clinician, the laboratory should issue an updated report with the appropriate variant classification.
**XI**	**Variant Interpretation in the Context of Clinical Significance** - As with standard-of-care biomarker testing, it is strongly recommended that pathologists participate in biomarker interpretation training and validation for new targetable alterations in NSCLC. - All variants in the report should be described at the cDNA and protein level, using Human Genome Variation Society nomenclature, and in relation to a reference transcript ID. In addition, nomenclature associated with treatment indications should be included, for example, EGFR T790M as well as NM_005228.4(EGFR):c.2369C > T(p.Thr790Met), if agreed upon by the multidisciplinary clinical team. - Variant annotation should include a description of the protein, the variant type, exon location if clinically relevant, and a brief summary of the clinical importance of the variant including expected responsiveness/resistance to therapies. - Reports may refer to a resource for finding information on clinical trials for which a patient may be eligible, rather than including a list of clinical trials.

## Supplementary Materials

The following supporting information can be downloaded at: https://www.mdpi.com/article/10.3390/curroncol29070396/s1, Supplemental Data S1: Summary of Content to Include on a Report from an NGS Panel Test. Supplementary Figure S1: Recommendations for Comprehensive Biomarker Testing in Advanced Non-Small Cell Lung Cancer.
