# Supplementary material for: Consensus Recommendations to Optimize Testing for New Targetable Alterations in Non-Small Cell Lung Cancer
Source: Curr Oncol. 2022 Jul 15;29(7):4981–97. doi: 10.3390/curroncol29070396 (PMC9318743; doi:10.3390/curroncol29070396)
Supplement: Supplementary file 1 [file curroncol-29-00396-s001.zip › curroncol-1784547-supplementary figure.pdf]

Recommendations for Comprehensive Biomarker Testing in Advanced Non-Small Cell Lung Cancer

Introduction:

A **biomarker is a measurable characteristic that provides information about a disease or condition.** The biomarkers discussed in this publication are alterations in genes that contribute to tumour growth and progression in patients with non-small cell lung cancer (NSCLC).

Biomarker testing in NSCLC helps match the right treatment to a patient's specific subtype of cancer.

Patients with certain biomarkers in their cancer can be treated with a targeted therapy that matches the biomarkers found in their cancer.

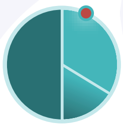

About 35 to 50% of patients with advanced non-squamous NSCLC have this type of biomarker.

Treatment with targeted therapy may prolong survival and result in a better quality of life for patients than chemotherapy because targeted therapies are typically associated with fewer side effects.

Many new targeted therapies are being developed and approved for use in NSCLC, with **23 new drug approvals in the past five years, compared to only 8 new approvals in the previous ten years**. Many of these new drugs are targeted therapies that require testing for new biomarkers.

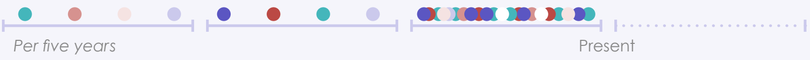

Overview:

This publication aims to provide guidance for the following:

1. **Oncologists,** to understand the **different technologies and their limitations** in testing for new biomarkers in NSCLC;
2. **Pathologists and laboratories,** to **optimize their testing methods** and ensure that **results are reported back to oncologists and patients clearly**; and
3. **Patients,** to gain awareness and understanding of **why biomarker testing is being done** and, if not already underway, **request testing** to support the best possible treatment outcomes.

Methods:

The recommendations in this manuscript were **developed by an expert working group consisting of physicians across the various specialties** involved in biomarker testing in lung cancer: oncology, pathology and clinical genetics.

The lead authors drafted recommendations based on published evidence and the recommendations were reviewed and revised by working group members in two virtual meetings. **Patient advisors reviewed the recommendations, provided their input, and collaborated to create the plain language summary.**

Results:

A key recommendation from the working group is that all patients with non-squamous NSCLC should receive comprehensive biomarker testing at diagnosis of NSCLC and again at progression.

Biomarker testing should be done as a reflex test, which means that when the pathologist diagnoses NSCLC, biomarker testing is initiated rather than requiring the oncologist to request biomarker testing.

Liquid biopsy (testing of biomarkers through a blood sample) can be used along with, or instead of, testing of biomarkers from a tissue biopsy. Liquid biopsy is preferred at progression because it may allow for the avoidance of a tissue biopsy and the potential resulting complications, it can provide faster biomarker results, and it is less costly. If a liquid biopsy is negative, a tissue biopsy should be attempted.

Comprehensive biomarker testing means that all biomarkers that are associated with a potential targeted therapy should be tested, including therapies that are already on the market, those anticipated to be available soon, and those for which a clinical trial may be available.

The working group also provided recommendations on the reporting biomarker testing results to ensure that they are clear for the treating clinician.

Conclusions:

It is important to **test for all biomarkers** that could affect treatment decisions in NSCLC.

In addition, it is critical that **biomarker testing is positioned to keep up with the pace of new drug development** in lung cancer.

These consensus recommendations are **intended as a guide to optimize testing** of new biomarkers in lung cancer.
