# Supplementary material for: Consensus Recommendations to Optimize Testing for New Targetable Alterations in Non-Small Cell Lung Cancer
Source: Curr Oncol. 2022 Jul 15;29(7):4981–97. doi: 10.3390/curroncol29070396 (PMC9318743; doi:10.3390/curroncol29070396)
Supplement: Supplementary file 1 [file curroncol-29-00396-s001.zip › curroncol-1784547-supplementary.pdf]

Current Oncology

Summary of Content to Include on a Report from an NGS Panel Test

Information on reports should follow provincial laboratory accreditation requirements. In the absence of such guidelines, at minimum reports should include information described below.

Case and Specimen Details

Reports should include patient identifiers (including name, DOB, biological sex) and specimen-specific details (including anatomic site, diagnosis, date collected). Name of the referring physician(s) must be included.

Results and Interpretation

Variants reported should be properly annotated using HGVS nomenclature, and where needed colloquial names to aid in comprehension by the end user. It is recommended to include allele frequency and copy numbers for each SNV and CNV, respectively. Classification and/or interpretation statements pertaining to each variant should be included and should follow standardized guidelines, such as Li et al [1]. Variants of strong/potential clinical significance and uncertain significance should be included on the report. Variants that are not clinically significant (i.e. benign variants) should be excluded.

Methodologies

Methods should include a description of the assays used for DNA/RNA extraction, library construction and sequencing, and bioinformatics tools/software used in the analysis of the sequencing data (e.g. alignment and variant calling tools). Sensitivity and specificity of the assay, the regions covered, and the minimum lower limit of detection should be included. The version of genome build used should be included.

Regions of low coverage, gene lists, and preferred transcript lists should be made available upon request.

References

1. Li, M.M.; Datto, M.; Duncavage, E.J.; Kulkarni, S.; Lindeman, N.I.; Roy, S.; Tsimberidou, A.M.; Vnencak-Jones, C.L.; Wolff, D.J.; Younes, A., et al. Standards and Guidelines for the Interpretation and Reporting of Sequence Variants in Cancer: A Joint Consensus Recommendation of the Association for Molecular Pathology, American Society of Clinical Oncology, and College of American Pathologists. *J Mol Diagn* **2017**, *19*, 4-23, doi:10.1016/j.jmoldx.2016.10.002.
